# Retrospective study for correlation analysis of nutritional status with osteoporosis, sarcopenia and cognitive impairment in elderly patients with coronary heart disease

**DOI:** 10.3389/fcvm.2023.1335572

**Published:** 2024-02-01

**Authors:** Xiao Xu, Daohong Li, Shan Zhang

**Affiliations:** Department of Geriatric, Suzhou Ninth People’s Hospital, Suzhou, Jiangsu, China

**Keywords:** coronary heart disease, nutritional risk index for the elderly, osteoporosis, sarcopenia, cognitive impairment

## Abstract

Coronary heart disease (CHD) is an abbreviation of coronary atherosclerotic heart disease, which remains challenging for diagnosis and treatment. Current study aims to explore the correlation between geriatric nutritional risk index (GNRI) and osteoporosis, sarcopenia, cognitive dysfunction in elderly patients with CHD, and to analyze the clinical diagnostic value of GNRI in the above complications. A total of 92 elderly patients with CHD treated in Suzhou Ninth People's Hospital from January 2020 to October 2023 were retrospectively collected as the experimental group, and 68 non-CHD subjects matched for sex and age in the same period of physical examination were randomly selected as the control group. Osteoporosis, sarcopenia and cognitive dysfunction were analyzed in all patients, and the correlation between GNRI and these indices in different populations was analyzed by Spearman's rank correlation. The diagnostic efficacy of GNRI in osteoporosis, sarcopenia, and cognitive impairment was analyzed by ROC curves. There was no significant difference in age, sex distribution, body mass index (BMI) and serum biological indexes between the elderly patients with CHD and the control group (all *P *> 0.05). Correlation analysis showed that GNRI level was positively correlated with bone mineral content (BMC), bone mineral density (BMD) T value and osteocalcin (OCN) (All *r* > 0, *P* < 0.05). In addition, GNRI levels were positively correlated with skeletal muscle mass (ASMI), grip strength and calf circumference (CC) (All *r* > 0, *P *< 0.05). However, there was no significant correlation between GNRI levels and cognitive dysfunction-related indicators (*P *> 0.05). In the elderly and elderly with CHD, the diagnostic AUC of GNRI was 0.875 and 0.862 in osteoporosis, and 0.912 and 0.932 in sarcopenia, respectively. The level of GNRI is significantly correlated with osteoporosis and sarcopenia. GNRI level, as an auxiliary diagnostic tool in elderly patients with CHD, exerts important clinical significance for early detection of the risk of complications, such as osteoporosis and sarcopenia.

## 1 Introduction

Coronary heart disease (CHD) is an abbreviation of coronary atherosclerotic heart disease, which is a heart disease caused by stenosis, spasm and even occlusion of the vascular lumen due to atherosclerotic lesions of coronary arteries, resulting in myocardial ischemia, hypoxia or necrosis (1). In recent years, the incidence of CHD has increased year by year, seriously endangering human health (2). Elderly patients with CHD are in a state of cardiac insufficiency for a long time, physiology and metabolism are affected to some extent, and are prone to malnutrition (3). Malnutrition is a common comorbidity in elderly patients, particularly in the elderly population, with adverse effects on the short- and long-term prognosis of patients (4). Nutritional status is affected in many ways, and poor nutritional status can exacerbate the severity of the disease in patients (5). Malnutrition and CHD interact to affect patient outcomes (5). Studies evaluating the nutritional status of patients have shown that elderly patients with poor nutritional status are higher than those with normal nutritional status in terms of infection incidence, case fatality, length of stay and medical costs (6).

Malnutrition in elderly patients with CHD predisposes to other complications (6). Osteoporosis is more common in the elderly, characterized by increased bone fragility and decreased bone mineral density (7). The etiology of osteoporosis is complex, among which malnutrition is an important cause, while the elderly are more prone to malnutrition due to physiological decline, poor dietary compliance and many underlying diseases (8). In addition, malnutrition is extremely common in elderly patients, mainly manifested by loss of muscle content and thus predisposes to the development of sarcopenia (9). Sarcopenia is the most common health problem in the elderly population and is one of the important causes leading to falls, fractures and decreased cardiopulmonary function in the elderly (9). Since the onset of sarcopenia is insidious and there are no obvious clinical symptoms in the early stage, most patients with sarcopenia are not actively diagnosed and treated (10). Early screening for sarcopenia and timely intervention are beneficial to improve clinical outcomes in elderly patients with CHD (11). In addition, sarcopenia may be related to cardiac function in patients, and chronic heart failure is a risk factor for the development of sarcopenia (12). Furthermore, CHD is tightly associated with the development of cognitive impairment, with an average 45% increased the risks of cognitive impairment or dementia in patients with CHD (13, 14). Studies by Greaves et al. showed that approximately 40% of patients with CHD undergoing cardiac bypass surgery were diagnosed with cognitive impairment within 1–5 years after surgery (15). Cognitive impairment occurred in 42.4% of patients with stable CHD after 4 years of follow-up (16).

Analyzing the correlation between malnutrition and osteoporosis, sarcopenia, cognitive impairment in elderly patients with CHD is helpful to intervene pertinently and reduce the occurrence and development of the above complications in elderly patients with CHD. However, studies on malnutrition in elderly patients with CHD have focused on patients with diabetes mellitus, lung infection, tumor, or perioperative period (17, 18), and studies on nutritional status in elderly patients with osteoporosis, sarcopenia, and cognitive impairment are lacking.

In this study, we try to analyze the relationship between nutritional status and osteoporosis, sarcopenia and cognitive dysfunction in elderly patients with CHD. At the same time, the predictive value of nutritional status on complications, such as osteoporosis, sarcopenia and cognitive impairment in elderly patients with CHD, was also explored in order to provide theoretical basis for early intervention.

## 2 Data and methods

### 2.1 Clinical data

**Figure 1:**
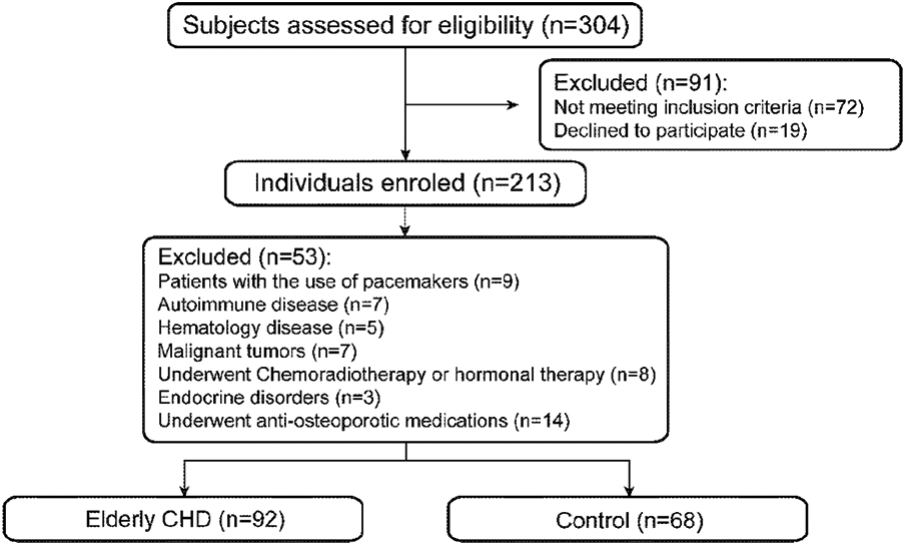
Flow diagram illustrating selection of study participants.

A total of 92 elderly patients with CHD treated in Suzhou Ninth People’s Hospital from January 2020 to October 2023 were retrospectively collected as the experimental group, and 68 non-CHD subjects matched for sex and age in the same period of physical examination were randomly selected as the control group (Figure 1).

Inclusion criteria of case group: (1) Age ≥60 years old; (2) The patients met the diagnostic criteria of CHD and were diagnosed as CHD by coronary angiography; (3) The clinical data of the patients are complete; (4) Sign the informed consent form.

Inclusion criteria of control group: (1) Age ≥60 years old; (2) Patients did not meet the diagnostic criteria of CHD; (3) The clinical data of the patients are complete; (4) Sign the informed consent form.

Exclusion criteria: (1) Atrial fibrillation, supraventricular tachycardia, pacemaker pacing rhythm; (2) Patients with autoimmune diseases and severe acute and chronic infectious diseases; (3) Patients with malignant tumors and hematological diseases; (4) End-stage elderly patients who have received radiotherapy, chemotherapy and hormone therapy; (5) Endocrine diseases such as hypothyroidism and hyperthyroidism, long-term use of glucocorticoids or surgery may affect bone mineral density; (6) Patients with routine anti-osteoporosis treatment.

### 2.2 Methods

#### 2.2.1 Examination of general clinical data

General data such as sex, age and blood pressure of the two groups were collected. Height and weight were measured and BMI was calculated. Venous blood samples were collected overnight for more than 10 h, and metabolic parameters including fasting blood glucose level (FBGL), total cholesterol (TC), triglyceride (TG), high density lipoprotein cholesterol (HDL-C), low density lipoprotein cholesterol (LDL-C) and serum albumin were measured by Siemens ADVIA 2400 automatic biochemical analyzer.

#### 2.2.2 Bone metabolism parameters

BMC and BMD T value were measured with Lunar-DPX-MD DXA from GE company. The T value of BMD of L1-L4 vertebrae in each patient was measured in anteroposterior position. According to WHO diagnostic criteria, osteoporosis is diagnosed if the T value of bone mineral density is ≤−2.5 SD (19). The serum OCN levels were measured by enzyme-linked immunosorbent assay (ELISA) method. The parameters of bone metabolism, including procollagen of type I N-propeptide (P1NP), β-isomerized C-terminal telopeptides (β-CTX), 25-hydroxyvitamin D (25-OH-D) and parathyroid hormone (PTH), were measured by Swiss Roche electrochemiluminescence analyzer Cobas e602.Geriatric Nutritional Risk Index (GNRI) = 1.489 × serum albumin + 41.7 × (body weight/ideal body weight). Male ideal weight = 0.75 × height-62.5, female ideal weight = 0.6 × height-40.

#### 2.2.3 Examination of metabolic parameters in sarcopenia

Skeletal muscle mass (ASM) and percent total body fat (TBF%) of the extremities were measured using GE Lunar-DPX-MD DXA. Skeletal muscle mass index (ASMI) of extremities = ASM/Height^2^. Grip strength and gait speed were used to measure muscle strength and area-provided energy, respectively. Grip strength was tested by grip strength meter CamryEH101. All subjects underwent a 6-m reentrant movement measurement of gait speed. The calf circumference (CC) was measured with an inelastic band at a resolution of 1 mm, measured by researchers trained in standardized measurements. Waist to hip ratio (WHR) = waist/hip circumference.

#### 2.2.4 Cognitive function test

The subjects’ overall cognitive function was assessed using the Mini-Mental State Examination (MMSE) and Montreal Cognitive Assessment Scale (MoCA). The MMSE and MoCA scale assessments were completed by two deputy chief physicians. The MMSE scores included language, calculation, visuospatial, place orientation, time orientation, attention, immediate memory, delayed memory and so on, with a total of 30 items, with a total score of 30. The score was positively correlated with the level of cognitive status, and MMSE <27 was judged as cognitive impairment. The MoCA scale included 8 cognitive assessments of visuospatial executive ability, naming, memory, attention, language fluency, abstract thinking, delayed memory, orientation and so on, with a total of 30 points. The score was positively correlated with the level of cognitive status, and MoCA <27 was judged as cognitive impairment.

### 2.3 Statistical analysis

The data were analyzed by SPSS 27.0 software. The measurement data of normal distribution are expressed as x ± s, and the data of normal distribution are expressed as medians (first quartile, third quartile). Independent sample *t*-test was used for comparison between the two groups. Counting data were expressed by number and percentage, and *χ*2 test was used for comparison between the two groups. Correlation analysis was performed by Spearman rank correlation. Diagnostic power analysis was performed by ROC curves. *P *< 0.05 was statistically significant.

## 3 Results

### 3.1 Analysis of general data of elderly patients with CHD

According to the inclusion and exclusion criteria, 92 elderly patients with CHD and 68 controls were included in this study. There were 38 males and 54 females in the elderly patients with CHD, while 33 males and 35 females in the control group. There was no significant difference in the sex ratio between the two groups (*P *> 0.05). The results showed that systolic blood pressure (SBP), diastolic blood pressure (DBP) and fasting blood glucose level (FBGL) were non-normal distribution, and other data obeyed normal distribution. Mann–Whitney *U* rank sum test (statistic is Z) or independent sample t test (statistic is t) will be performed for measurement data, and the results are shown in Table 1. The height and weight of the elderly patients with CHD were significantly lower than those of the control group (all *P *< 0.01). In addition, there were no significant differences in age, BMI, blood pressure, lipid-related indexes, serum albumin level and FBGL between the elderly patients with CHD and the control group (all *P *> 0.05).

**Table 1:** Comparison of general data between elderly patients with CHD and control subjects.

Characteristic	Elderly CHD group (*n* = 92)	Control group (*n* = 68)	t/Z	*P*
Sex (male/female)	38/54	33/35	0.909	0.363
Age	71.71 ± 7.37	70.04 ± 4.57	1.755	0.081
Height	162.25 ± 7.10	166.72 ± 5.70	−4.412	<0.001
Body weight	57.18 ± 9.62	61.13 ± 5.52	−3.273	0.001
BMI	21.68 ± 3.17	22.01 ± 1.97	−0.813	0.448
Systolic blood pressure	116.00 (105.25, 129.00)	116.00 (103.00, 130.75)	−0.116	0.908
Diastolic blood pressure	74.00 (68.00, 82.00)	77.00 (68.25, 84.75)	−0.884	0.377
TG (mmol/L)	1.54 ± 0.69	1.76 ± 0.70	−1.977	0.050
TC (mmol/L)	4.41 ± 1.23	4.24 ± 1.14	0.893	0.373
HDL-C (mmol/L)	1.20 ± 0.21	1.22 ± 0.23	−0.481	0.632
LDL-C (mmol/L)	2.53 ± 0.80	2.48 ± 0.70	0.414	0.679
Serum albumin (g/L)	32.95 ± 5.38	32.66 ± 6.53	0.304	0.762
FBGL (mmol/L)	5.40 (5.10, 5.80)	5.50 (5.00, 6.08)	−0.832	0.406

### 3.2 Relationship between nutritional status and osteoporosis in elderly patients with CHD

Spearman rank correlation was used to analyze the correlation between GNRI and different osteoporosis-related indexes. As shown in Table 2, GNRI in the total population was positively correlated with BMC, BMD T value, OCN and P1NP (All *r* > 0, *P* < 0.05), and negatively correlated with β-CTX and PTH (All *r* < 0, *P *< 0.05). Similarly, GNRI was positively correlated with BMC, BMD T value, OCN and P1NP (All *r* > 0, *P *< 0.05), but negatively correlated with β-CTX and PTH (All *r* < 0, *P *< 0.01).In addition, GNRI did not show significant correlations with P1NP or β-CTX in the control population (both *P *> 0.05). These results collectively suggested that the nutritional status was significantly correlated with the related indexes of osteoporosis.

**Table 2:** Correlation analysis between GNRI and osteoporosis-related indicators.

Variables	Total population		Elderly CHD group		Control group	
	*r*	*P*	*r*	*P*	*r*	*P*
BMC	0.421	<0.001	0.435	<0.001	0.409	<0.001
BMD T value	0.475	<0.001	0.439	<0.001	0.554	<0.001
OCN	0.503	<0.001	0.559	<0.001	0.420	<0.001
P1NP	0.170	0.031	0.268	0.010	0.009	0.944
β-CTX	−0.172	0.029	−0.307	0.003	0.037	0.762
25-OH-D	0.012	0.885	0.043	0.683	−0.036	0.770
PTH	−0.347	<0.001	−0.341	<0.001	−0.353	0.003

### 3.3 Relationship between nutritional status and sarcopenia in elderly patients with CHD

Spearman rank correlation was used to analyze the correlation between GNRI and different sarcopenia-related indicators. As shown in Table 3, there was a significant positive correlation between GNRI and ASMI, grip strength and CC in the total population (All *r* > 0, *P* < 0.05), but not with gait speed, WHR and TBF% (All *P* > 0.05). In addition, GNRI was positively correlated with grip strength, gait speed and CC (All *r* > 0, *P* < 0.05), but not with ASMI, WHR and TBF% (All *P* > 0.05). Interestingly, in the control population, GNRI showed a significant positive correlation only with ASMI (*r* > 0, *P* < 0.05), but not with other sarcopenia-related indicators (all *P* > 0.05).

**Table 3:** Correlation between GNRI and sarcopenia.

Variables	Total population		Elderly CHD group		Control group	
	*r*	*P*	*r*	*P*	*r*	*P*
ASMI	0.234	0.003	0.203	0.052	0.275	0.023
Grip strength	0.231	0.003	0.227	0.030	0.236	0.053
Gait speed	0.147	0.063	0.268	0.010	−0.052	0.671
CC	0.181	0.022	0.312	0.002	0.018	0.885
WHR	−0.003	0.972	−0.008	0.938	0.010	0.937
TBF%	0.071	0.374	−0.018	0.865	0.190	0.122

### 3.4 Relationship between nutritional status and cognitive impairment in elderly patients with CHD

Spearman's rank correlation was used to analyze the correlation between GNRI and different cognitive function-related indicators. As shown in Table 4, there was no significant correlation between GNRI and MMSE or Mo CA in the total population (All *P* > 0.05). Interestingly, there was a significant negative correlation between GNRI and Mo CA in the control population (*r *< 0, *P* < 0.05), possibly related to the small sample size. In addition, there was no significant correlation between GNRI and MMSE or MoCA scores in elderly patients with CHD (All *P* > 0.05).

**Table 4:** Correlation analysis between GNRI and different cognitive function-related indicators.

Variables	Total population		Elderly CHD group		Control group	
	*r*	*P*	*r*	*P*	*r*	*P*
MMSE	−0.048	0.547	−0.021	0.843	−0.118	0.337
MoCA	0.028	0.722	0.081	0.443	−0.240	0.048

### 3.5 Predictive value of GNRI in osteoporosis, sarcopenia or cognitive impairment in elderly patients with CHD

**Figure 2:**
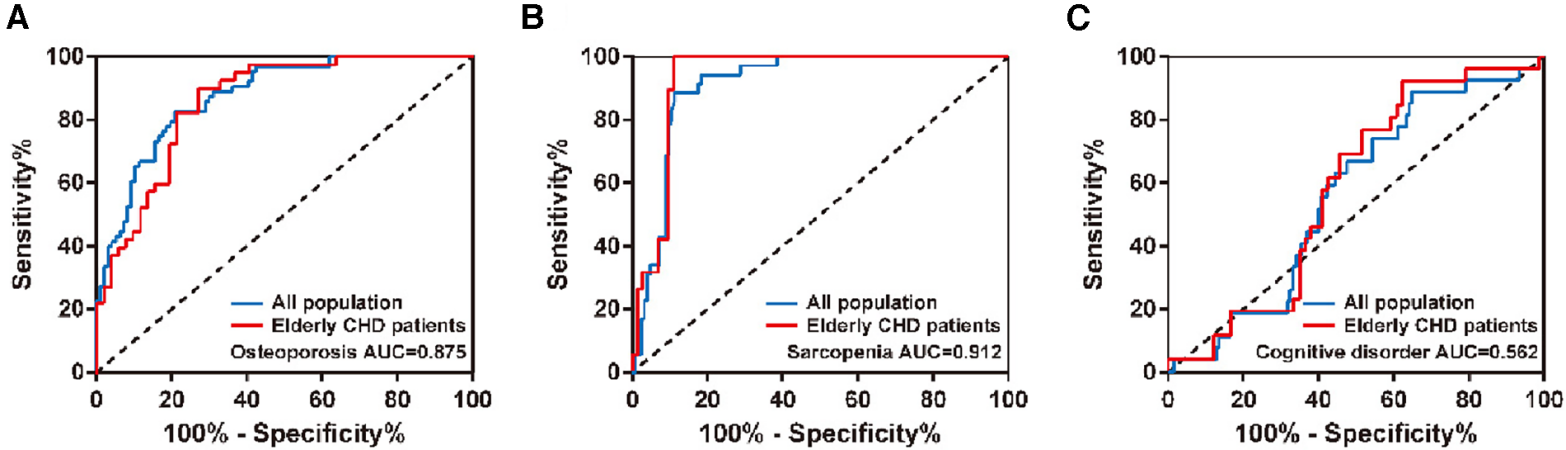
ROC curves of GNRI in the diagnosis of (**A**) osteoporosis, (**B**) sarcopenia, or (**C**) cognitive impairment in different populations.

The diagnostic efficacy of nutritional status in osteoporosis, sarcopenia, or cognitive impairment was analyzed by ROC curves in the total population and in the elderly with CHD.As shown in Figure 2 and Table 5, the diagnostic AUCs of GNRI for osteoporosis and sarcopenia in the total population were 0.875 and 0.912, respectively. In addition, the diagnostic sensitivity was 82.5% and 88.6%, and the diagnostic specificity was 79.4% and 88.8%, respectively. Similarly, GNRI showed high diagnostic AUC, sensitivity, and specificity in both osteoporosis and sarcopenia in the elderly population with CHD. In particular, when the Youden index was 0.890, the sensitivity of GNRI in the diagnosis of sarcopenia reached 100%. These results collectively suggested that GNRI has the potential to be an early diagnostic modality for osteoporosis and sarcopenia. In addition, the AUCs of GNRI in the diagnosis of cognitive impairment were only 0.562 and 0.583 in the total population and in the elderly population with CHD, respectively, indicating that the nutritional status of patients has no potential diagnostic value for cognitive impairment.

**Table 5:** Diagnostic efficacy of GNRI in osteoporosis, sarcopenia, or cognitive impairment.

Population	Indication	AUC (95% CI)	Youden index	Sensitivity (%)	Specificity (%)	*P* value
Total population	Osteoporosis	0.875 (0.823, 0.928)	0.619	82.5	79.4	<0.001
	Sarcopenia	0.912 (0.866, 0.957)	0.774	88.6	88.8	<0.001
	Cognitive disorder	0.562 (0.457, 0.667)	0.242	88.9	35.3	0.311
Elderly CHD patients	Osteoporosis	0.862 (0.788, 0.935)	0.631	90.0	73.1	<0.001
	Sarcopenia	0.932 (0.880, 0.983)	0.890	100.0	89.0	<0.001
	Cognitive disorder	0.583 (0.464, 0.702)	0.302	92.3	37.9	0.218

## 4 Discussion

CHD is a heart disease caused by a variety of factors leading to atherosclerosis of coronary arteries, leading to stenosis or even occlusion of the vascular lumen, followed by myocardial hypoxia, ischemia or necrosis (2). In recent years, the incidence of cardiovascular diseases is still rising sharply, about 2.5% worldwide. Nine billion people suffer from cardiovascular disease, 1.1 million of whom are patients with CHD (20). With the development of medical level and improvement of surgical technical concept, the mortality and complication rate of CHD in the elderly have been greatly reduced, but risk factors need to be strictly controlled to improve the prognosis (21). The poor nutritional status of elderly patients with CHD is related to aging, cardiac insufficiency, malabsorption of nutrients due to gastrointestinal congestion, multiple concomitant underlying diseases and polypharmacy (22). In recent years, more studies have shown that nutritional status is associated with the prognosis of CHD (23). In addition, malnutrition is prone to the occurrence of various prognostic complications, such as osteoporosis, sarcopenia and cognitive impairment. The GNRI is a nutritional assessment index based on serum albumin and BMI. GNRIs are primarily used to assess the risk of complications associated with malnutrition in the elderly population (24). Unlike other nutritional indicators, GNRI is obtained by calculation of albumin, height, and weight, is easy to perform, is less affected by subjective factors, and has good inter-rater agreement (25). GNRI was first proposed by BOWEN et al. in 2005 to predict malnutrition-related complications (pressure ulcers and infections) and mortality in hospitalized elderly patients (26).

Therefore, the purpose of this study was to investigate the correlation between GNRI and osteoporosis, sarcopenia, cognitive dysfunction in elderly patients with CHD, and to analyze the diagnostic efficacy of GNRI in osteoporosis, sarcopenia and cognitive dysfunction by ROC curve.

A total of 92 elderly patients with CHD and 68 control patients were included in this study. Firstly, the clinical data of the two groups were analyzed. There was no significant difference in age distribution and sex composition between the elderly patients with CHD and the control group (*P* > 0.05). In addition, the height and weight of the elderly patients with CHD were significantly lower than those of the control group (both *P *< 0.01), but the BMI level was not significantly different between the two groups (*P *> 0.05). In addition, there were no significant differences in age, BMI, blood pressure, lipid-related indexes, serum albumin level and FBGL between the elderly patients with CHD and the control group (all *P *> 0.05). There was no significant difference in the general clinical data between the elderly patients with CHD and the control group, indicating the feasibility of the study. Therefore, we further analyzed the association of GNRI with osteoporosis, sarcopenia, and cognitive impairment in different populations.

Due to the decline of body function, poor digestive and absorption function, the incidence of malnutrition is high. Malnutrition can lead to insufficient absorption of trace elements such as calcium, phosphorus, zinc, magnesium, copper, imbalance of bone metabolism, and lead to osteoporosis. The results of Spearman's rank correlation analysis showed that GNRI was positively correlated with BMC, BMD T value, OCN and P1NP, but negatively correlated with β-CTX and PTH, suggesting that the nutritional status of the elderly population was significantly correlated with the occurrence and development of osteoporosis. In recent years, many studies have found a close relationship between CHD and osteoporosis (27, 28). Samelson et al. have shown a higher incidence of CHD in women with low bone mineral density (28). Marcovitz et al. used coronary angiography to evaluate the degree of coronary stenosis in 209 patients. Current results showed that osteoporosis or osteopenia were independent risk factors for CHD in the elderly population, and the highest relative risk of osteoporosis was found in multivariate analysis of traditional risk factors of osteoporosis and CHD (e.g., diabetes mellitus, hypertension, smoking, hyperlipidemia, family history of cardiovascular disease, etc.) (27, 28). In this study, both GNRI and osteoporosis-related indicators in the elderly population with CHD showed significant correlations, consistent with previous findings (27, 28).

Previous studies have confirmed that nutritional factors are closely related to osteoporosis, as well as nutrition-related diseases such as sarcopenia, and that sarcopenia and osteoporosis share many of the same pathogenesis (29, 30). In addition, studies have shown that nutrient deficiency and malnutrition are also important risk factors for sarcopenia, such as vitamin D, vitamin A and mineral deficiencies (31). In this study, GNRI was positively correlated with ASMI, grip strength, CC and other sarcopenia-related indicators in the total population, suggesting that the nutritional status of the elderly population is significantly correlated with the occurrence of sarcopenia. Because elderly people are also at high risk for cardiovascular disease, comorbidities of sarcopenia and cardiovascular disease are common. Sarcopenia is an important complication of abnormal cardiac functions, which in turn can accelerate the occurrence of sarcopenia (32). Sarcopenia has been shown to be an independent predictor of decreased cardiac function in patients with chronic heart failure (33). Chronic heart failure can contribute to the development of sarcopenia through a variety of pathophysiological mechanisms, including malnutrition and inflammation (34). The results of Spearman's rank correlation analysis showed that GNRI was significantly correlated with sarcopenia-related indexes such as grip strength, gait speed and CC in elderly patients with CHD. We hypothesize that as nutritional risk increases in patients with CHD, heart failure progresses and cardiac function further declines, and skeletal muscle mass progresses to sarcopenia. The occurrence of sarcopenia may be due to decreased muscle strength and affect motor function, resulting in dyspnea and decreased cardiac function, further exacerbating heart failure.

There are few studies on the association between nutritional status and cognitive impairment. Some studies have suggested that nutritional status predicts mortality and the probability of complications in patients with mild cognitive impairment (35, 36). In addition, some studies have shown a 23% risk of malnutrition in patients with mild cognitive impairment (35). At the same time, malnutrition can also promote negative emotions, exacerbate their cognitive impairment and reduce their quality of life (36). However, the results of this study showed no significant correlation between GNRI and cognitive function-related measures in the elderly or elderly with CHD, suggesting no potential link between nutritional status and the development of cognitive dysfunctions in patients.

GNRI was associated with osteoporosis and sarcopenia. Thus, osteoporosis and sarcopenia can be predicted by GNRI. Therefore, we analyzed the diagnostic efficacy of GNRI for osteoporosis, sarcopenia, and cognitive impairment in different populations by ROC curves. GNRI predicted the largest area under the ROC curve for sarcopenia, both in the elderly and in the elderly with CHD. In addition, the sensitivity of GNRI in the diagnosis of sarcopenia reached 100% in elderly patients with CHD, indicating that there were no missed cases. In addition, GNRI also showed high diagnostic efficacy for osteoporosis in different populations. From the point of view of human anatomy, skeleton and muscle are adjacent. Common factors regulating osteoporosis and sarcopenia include nutritional factors, genetic factors, endocrine factors and disease factors, and they have closely related signaling pathways and common targets. This may also account for the similar diagnostic efficacy of GNRI in osteoporosis and sarcopenia. In addition, the low AUC of GNRI in the diagnosis of cognitive impairment in different populations suggests that the nutritional status of patients has no potential diagnostic value for cognitive impairment.

The study has the following limitations: (1) This study is a single-center, retrospective, observational study, which is limited to some extent by the clinical data of patients, and inaccurate description will interfere with the results. For the inaccurate data recorded, it may lead to the deviation of one item in CHD group or control group, further resulting in the difference of this indicator or inaccurate correlation analysis results; (2) The sample size is relatively small, and there is some bias in the process of retrospective data collection; (3) At present, there are few reports on this topic, so it is difficult to compare with other studies of the same type and analyze the similarities and differences between different studies.

In conclusion, the nutritional status in elderly patients with CHD is significantly associated with the development of osteoporosis and sarcopenia, with higher nutritional risk as well as the higher risk indicators-related to osteoporosis and sarcopenia. GNRI can be used as an early diagnostic modality for the complications of osteoporosis and sarcopenia in elderly patients with CHD, and has important significance in improving the clinical prognosis of CHD and preventing the occurrence of osteoporosis and sarcopenia.

## Data Availability

The raw data supporting the conclusions of this article will be made available by the authors, without undue reservation.
